# Unsupervised natural language processing in the identification of patients with suspected COVID-19 infection

**DOI:** 10.1590/0102-311XEN243722

**Published:** 2023-12-04

**Authors:** Rildo Pinto da Silva, Juliana Tarossi Pollettini, Antonio Pazin

**Affiliations:** 1 Faculdade de Medicina de Ribeirão Preto, Universidade de São Paulo, Ribeirão Preto, Brasil.

**Keywords:** COVID-19, Natural Language Processing, Health Care, Selection Criteria, Proprietary Health Facilities, COVID-19, Processamento de Linguagem Natural, Atenção à Saúde, Critérios de Seleção de Pacientes, Instituições Privadas de Saúde, COVID-19, Procesamiento de Lenguaje Natural, Atención a la Salud, Criterios de Seleción de Pacientes, Instituciones Privadas de Salud

## Abstract

Patients with post-COVID-19 syndrome benefit from health promotion programs. Their rapid identification is important for the cost-effective use of these programs. Traditional identification techniques perform poorly especially in pandemics. A descriptive observational study was carried out using 105,008 prior authorizations paid by a private health care provider with the application of an unsupervised natural language processing method by topic modeling to identify patients suspected of being infected by COVID-19. A total of 6 models were generated: 3 using the BERTopic algorithm and 3 Word2Vec models. The BERTopic model automatically creates disease groups. In the Word2Vec model, manual analysis of the first 100 cases of each topic was necessary to define the topics related to COVID-19. The BERTopic model with more than 1,000 authorizations per topic without word treatment selected more severe patients - average cost per prior authorizations paid of BRL 10,206 and total expenditure of BRL 20.3 million (5.4%) in 1,987 prior authorizations (1.9%). It had 70% accuracy compared to human analysis and 20% of cases with potential interest, all subject to analysis for inclusion in a health promotion program. It had an important loss of cases when compared to the traditional research model with structured language and identified other groups of diseases - orthopedic, mental and cancer. The BERTopic model served as an exploratory method to be used in case labeling and subsequent application in supervised models. The automatic identification of other diseases raises ethical questions about the treatment of health information by machine learning.

## Abstract (pt)

Os pacientes com síndrome pós-COVID-19 se beneficiam de programas de promoção de saúde e sua rápida identificação é importante para a utilização custo efetiva desses programas. Técnicas tradicionais de identificação têm fraco desempenho, especialmente em pandemias. Portanto, foi realizado um estudo observacional descritivo utilizando 105.008 autorizações prévias pagas por operadora privada de saúde com aplicação de método não supervisionado de processamento de linguagem natural por modelagem de tópicos para identificação de pacientes suspeitos de infecção por COVID-19. Foram gerados seis modelos: três utilizando o algoritmo BERTopic e três modelos Word2Vec. O modelo BERTopic cria automaticamente grupos de doenças. Já no modelo Word2Vec, para definição dos tópicos relacionados a COVID-19, foi necessária análise manual dos 100 primeiros casos de cada tópico. O modelo BERTopic com mais de 1.000 autorizações por tópico sem tratamento de palavras selecionou pacientes mais graves - custo médio por autorizações prévias pagas de BRL 10.206 e gasto total de BRL 20,3 milhões (5,4%) em 1.987 autorizações prévias (1,9%). Teve 70% de acerto comparado à análise humana e 20% de casos com potencial interesse, todos passíveis de análise para inclusão em programa de promoção à saúde. Teve perda importante de casos quando comparado ao modelo tradicional de pesquisa com linguagem estruturada e identificou outros grupos de doenças - ortopédicas, mentais e câncer. O modelo BERTopic serviu como método exploratório a ser utilizado na rotulagem de casos e posterior aplicação em modelos supervisionados. A identificação automática de outras doenças levanta questionamentos éticos sobre o tratamento de informações em saúde por aprendizado de máquina.

## Abstract (es)

Los pacientes con síndrome pos-COVID-19 pueden beneficiarse de los programas de promoción de la salud. Su rápida identificación es importante para el uso efectivo de estos programas. Las técnicas de identificación tradicionales no tienen un buen desempeño, especialmente en pandemias. Se realizó un estudio observacional descriptivo, con el uso de 105.008 autorizaciones previas pagadas por un operador de salud privado mediante la aplicación de un método no supervisado de procesamiento del lenguaje natural mediante modelado temático para identificar a los pacientes sospechosos de estar infectados por COVID-19. Se generaron 6 modelos: 3 con el uso del algoritmo BERTopic y 3 modelos Word2Vec. El modelo BERTopic crea automáticamente grupos de enfermedades. En el modelo Word2Vec para definir temas relacionados con la COVID-19, fue necesario el análisis manual de los primeros 100 casos de cada tema. El modelo BERTopic con más de 1.000 autorizaciones por tema sin tratamiento de palabras seleccionó a pacientes más graves: costo promedio por autorizaciones previas pagada de BRL 10.206 y gasto total de BRL 20,3 millones (5,4%) en 1.987 autorizaciones previas (1,9%). Además, contó con el 70% de aciertos en comparación con el análisis humano y el 20% de los casos con potencial interés, todos los cuales pueden analizarse para su inclusión en un programa de promoción de la salud. Hubo una pérdida significativa de casos en comparación con el modelo tradicional de investigación con lenguaje estructurado y se identificó otros grupos de enfermedades: ortopédicas, mentales y cáncer. El modelo BERTopic sirvió como un método exploratorio para ser utilizado en el etiquetado de casos y su posterior aplicación en modelos supervisados. La identificación automática de otras enfermedades plantea preguntas éticas sobre el tratamiento de la información de salud mediante el aprendizaje de máquina.

## Introduction

The COVID-19 ^1^ pandemic reinforced the historical concern of researchers regarding the threat of new viruses and mutation of existing ones. It implied pressure on already overburdened health care services ^2^, by severe forms of the disease (approximately 25% of vulnerable patients or patients with comorbidities) and a high mortality rate (5.6% in the firstwave ^3^). Additionally, structural changes in health care services, greater impact on low- and middle-income countries ^4^, ethical conflicts in the prioritization of care ^5^ and financial challenges accentuated their impact. Challenges were aggravated by the emergence of long COVID-19 or post-COVID syndrome ^6^ ^,^ ^7^, which affects 10% to 30% of patients ^8^. New pandemics are expected to emerge in the future ^9^ and early identification of patients will be important for correct and cost-effective adoption of care.

The treatment of information is a challenge, due to its increasing volume ^10^ or due to the peculiarities of the different areas of knowledge. In health care, data are incomplete, heterogeneous, multidimensional, unstructured and inaccurate ^11^ ^,^ ^12^. To address these challenges, it was proposed the discovery of knowledge through KDD (knowledge discovery in database) in the mining (data mining) of large volumes of data (big data) ^13^ ^,^ ^14^.

Machine learning (ML) techniques enable the algorithm to learn patterns that are unidentifiable by classification or prediction techniques ^15^. This learning can be supervised - with labels that classify the object of study - or unsupervised - with no classification. In this case, exploratory techniques are used for the creation of labels and subsequent application of supervised techniques ^15^. The labeling of medical data is difficult and depends on specialized work, being a limiting factor in studies of the pandemic ^16^. Thus, unsupervised exploratory techniques are an important step in the application of ML on large volumes of data for knowledge discovery.

Text data mining refers to the discovery of patterns as proposed by Fayyad et al. ^10^, while natural language processing (NLP) is seen as a branch of artificial intelligence that deals with human language ^17^ or makes this language understandable to computers ^18^ thereby enabling different approaches, including the grouping of texts by topics (“topic modeling”). Topics are groups of similar objects, being a particular case of clustering.

Health care providers process data necessary for regulatory ^19^ and health care cohesion. Among them, prior authorization is the process of verifying the eligibility of patients and the coherence between the disease and treatment. It is requested before health care. This process is indirectly regulated by the Brazilian National Supplementary Health Agency (ANS) by guaranteeing service deadlines ^20^.

Prior authorization analysis provides an opportunity for early patient selection. However, due to medical confidentiality, there is no information on the International Classification of Diseases, 10^th^ revision (ICD-10). Also, the requested care procedures do not allow the correct correlation with the disease to be treated and the complementary information of the prior authorization is not structured. Therefore, there is an opportunity for innovative solutions in the identification of patients in health care providers in Brazil. This is an important economic sector that covers approximately 25% of the Brazilian population with expenditures equivalent to 5.7% of gross domestic product (GDP) ^21^.

There are few studies using NLP in health care in Brazil. Duval et al. ^22^ built a pharmacosurveillance system using twitter to detect adverse events caused by drugs - they used as a model the drug doxycycline for the treatment of malaria. Moreira et al. ^23^ proposed a hybrid model through which NLP created patient clusters using unstructured data. These clusters were incorporated into structured data, improving the accuracy of the diagnosis of patients with suspected dementia ^23^. Diniz et al. ^24^ created a mobile phone system to identify patients with suicidal ideation that allowed the individual quantification of moment-to-moment risk (“digital phenotyping”) enabling the action of health care professionals.

No studies using supplementary health care data were found, probably due to the difficulty of access to data in this health care sector, limited by barriers of professional and commercial secrecy. This study fills this gap and contributes to the application of ML methods in free software through a real case study.

The objective of this article is to describe an unsupervised NLP method to identify patients with suspected COVID-19 infection through the analysis of a real database of prior authorizations issued by a private health care provider in the auto-management mode of the State of São Paulo, Brazil.

## Methods

### Study design and population

This is a descriptive observational study, based on secondary data from prior authorizations of a private health care provider in the State of São Paulo, in the auto-management mode (operator). Prior authorizations are requested by health care providers or beneficiaries before consultations, examinations, hospitalizations and other elective procedures. Emergency care authorization is automatically released in compliance with the rules of the legislation. For hospitalizations, only one authorization is issued covering the entire period of hospitalization of the patient. The payment of care to the provider only occurs upon submission of the prior authorization.

The database studied is anonymized, however, each prior authorization is issued to a specific beneficiary and there is a one-to-one relationship between prior authorization and beneficiary. The proposed method selects authorizations that contain information about suspected COVID-19 infection, and therefore the selected authorizations are considered to represent a patient with suspected COVID-19 infection.

The health care provider had, in the period, 29,336 beneficiaries exposed, of which 14,663 (50%) were female and 28,820 (98.2%) resided in the State of São Paulo. The mean age of the group was 45 years.

### Database and variables studied

Each authorization contains a blank text field, “clinicalindication”, in which the reason or justification for the prior authorization request is indicated. Filling in this field is not mandatory. The provider may only attach documents justifying the request for the procedure. In this case, it is common to fill in the field with text “attached” or not to fill it in. The “clinicalindication” variable is the variable of interest in this study.

Prior authorizations issued between September 1^st^, 2019 and June 30, 2022 were selected (n = 742,901). Those missing the justification (missing values) in the “clinicalindication” field (n = 558,530, 75%) were excluded. Therefore, 184,371 (25%) prior authorizations were included in this study, of which 105,008 contain payment information. Each prior authorizations contains at least one health care event identified in the event structure and event description variables corresponding respectively to the code of the requested event and its description. Authorizations are classified according to: type (“treatmenttype”), regime (“treatmentregime”) and objective of care (“treatmentobjective”). Filling in the ICD-10 field is not mandatory. They have an expiration date (“expirationdate”) and can be canceled, reissued or revalidated according to the provider’s administrative criteria. Box 1 contains the variables present in the database and used in this study.

**Box 1:** Variables from the prior authorization database of a private health care provider. São Paulo, Brazil.

VARIABLE	VARIABLE DESCRIPTION	VARIABLE TYPE	VARIABLE TRANSFORMATION
authorization (prior authorization)	Number of prior authorization for each authorized procedure	Numerical	No
authorizationtype	Type of authorization according to TISS standard - consultation, removal, extension, hospitalization summary and SADT	Text	No
authorizationdate	Date of authorization issuance	Date	No
expirationdate	Authorization expiration date	Date	No
icd	International Classification of Diseases, 10th revision (ICD-10) related to the authorization informed by the requesting service provider, it is not a mandatory field	Text	No
icd_description	Description of the ICD-10 related to the authorization	Text	No
careregimen	Type of facility used for care according to provider classification - outpatient clinic, home care, day hospital care, hospitalization, and emergency room	Text	No
treatmenttype	Type of treatment - surgical, clinical, obstetric, pediatric, psychiatric, dental	Text	No
treatmentobjective	Treatment objective - diagnostic, palliative, preventive, restorative, therapeutic	Text	No
requestdate	Date of request for prior authorization	Date	No
eventstructure	Prior authorization event code. The service provider indicates the health care event they wish to perform, which is analyzed by the health care provider and authorized	Text	No
eventdescription	Description of the authorized event	Text	No
clinicalindication	Field informed by the service provider which contains the justification for requesting the procedure of the prior authorization. This field is not mandatory and is supported by other information submitted as attachments. It is a free text field without any kind of automatic validation	Text	Yes *

SADT: diagnostic and therapeutic support services; TISS: information exchange in supplementary health.

* The transformation of this variable is described in the text of the article. It is the variable of interest for natural language processing analysis.

### Natural language processing

Two NLP models were applied - BERTopic (https://maartengr.github.io/BERTopic/index.html) and Word2Vec - described briefly below.

#### BERTopic model

The BERTopic model is an unsupervised algorithm for vector-based topic modeling. Topic modeling is a mining method whose objective is to discover hidden patterns considering the context and classify the respective texts into similar groups ^25^ ^,^ ^26^, called topics.

Initially, each document, in this case prior authorizations, is converted to its vector representation (word embedding) using the *Bidirectional Encoder Representations from Transformers* (BERT) model. The dimensionality of this representation is reduced using the *Uniform Manifold Approximation and Projection for Dimension Reduction* (UMAP) technique and the *Density-Based Clustering Based on Hierarchical Density Estimates* (HDBSCAN) algorithm is applied to create topics of documents that are semantically similar ^27^. For the description of each topic, we used the term frequency - inverse document frequency (TF-IDF) ^28^ ^,^ ^29^ ^,^ ^30^ ^,^ ^31^ method. Documents not classified by the model are grouped into a specific topic containing outliers. In this work, the methods were applied through a free library based on Python ^28^ called BERTopic.

Two parameters were used to define the minimum number of authorizations in each topic created: 500 or more (BERTopic +500) and 1,000 or more (BERTopic +1,000) defined in the min_topic_size parameter of the model. Since it is an automatic model, the total number of topics created depends on this parameter. The language parameter was defined as multilingual for modeling the text in Portuguese and the vectorization model - *embedding_model* - as all-MiniLM-L6-v2, which is the standard of the model.

To identify the topics belonging to COVID-19, the *get_topic_info()* method of the model itself was used, which generates the automatic description of the topic.

#### Word2Vec model

Word2Vec is an NLP model that uses neural networks to learn the representation of words (word embedding) in a high-dimensional vector space, capable of capturing the semantic and syntactic context of words in a given text corpus. For the comparative analysis, we used the *continuous Bag-of-Words* ^32^ ^,^ ^33^ model of the Word2Vec algorithm. The texts of the “clinicalindication” variable were separated into words (*tokens*) using the NLTK library (Natural Language Toolkit - https://www.nltk.org/), on which we applied the Word2Vec algorithm from the Gensim library (https://pypi.org/project/gensim/), using a vector size equal to 300, recalculated considering their average and categorized into 20 clusters using the K-Means algorithm. These clusters were considered the topics of this model. This method does not automatically assign names to topics. To identify clusters with suspected cases of COVID-19 infection, each of the 20 clusters was manually analyzed by the main researcher. To this end, the first 100 authorizations classified in descending order of expenditure were selected in each cluster. Each text present in the clinical indication variable was analyzed and the respective cluster was classified, or not, in the COVID-19 group.

Each of the two models was applied to the descriptions - treated or not - contained in the prior authorization of the variable “clinicalindication”. The treatment of the variable is recommended to improve the performance of the Word2Vec model.

The treatment of the clinical indication variable occurred as follows: conversion of all words into lowercase, removal of stopwords in Portuguese, exclusion of most common words in health and exclusion of special characters. No accents or other features of Portuguese were replaced. The words COVID-19 and SARS-CoV-2 were turned into *covid*. The ICD-10-related words present in the clinical indication variable were also standardized. 

### Evaluation of the quality of the classification generated by the models

Thus, we reached 6 different types of models: BERTopic +500, BERTopic +1,000 and Word2Vec, each with and without text treatment of the clinical indication variable (treated and untreated).

To assess the quality of the classification, the main author analyzed the BERTopic +1,000 model because it presented the highest average cost per authorization. Thus, the first 100 authorizations classified as suspected or COVID-19-related events by this model were ordered in descending order of cost. The clinical indication text of each of these authorizations was manually analyzed by and classified it into classes of interest for study. This manual classification was compared to the automatic classification generated in this model.

For comparison with traditional *structured query language* (SQL) research methods, all prior authorizations containing the words *covid*, *sars*, *coronavirus* and *coronavírus* in uppercase or lowercase letters were selected and compared with the models generated using the authorization number as a binding index and identifying whether they were part of the groups identified as suspected COVID-19 infection.

### Prior authorization cost 

Prior authorization cost corresponds to the health care expenditures of each prior authorization. The payment basis contains the expenses paid to service providers net of disallowance. Costs were obtained using the prior authorization number as the connecting key.

The total amount paid corresponds to the sum of all expenses in the period from September 2019 to July 2022 found in the payment basis for each prior authorization. The number of authorizations paid corresponds to the count of authorizations with an amount spent per authorization greater than BRL 0.00.

The average cost per paid authorization corresponds to the ratio between authorization expenditure and the number of paid authorizations. In this study, the most severe cases were those with the highest average cost per prior authorization. Expenditures are presented in reais and without inflation adjustment.

Access to data was granted through a confidentiality and scientific cooperation agreement with the provider and approved by the Research Ethics Committee of Ribeirão Preto School of Medicine, São Paulo University (HCFMUSP/RP; protocol n. 55685722.9.0000.5440).

## Results

**Figure 1:**
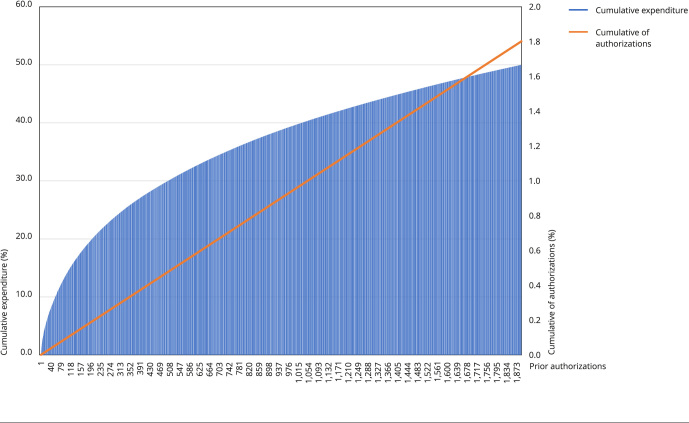
Cumulative percentage expenditure (up to 50%) and cumulative percentage of prior authorizations (%) of supplementary health provider. São Paulo, Brazil, September/2019 to June/2022.

A total of 742,901 authorizations were issued in the 34 months analyzed, of which 184,371 (24.9%) were filled in with at least one number or word, are part of this study and were analyzed. Of these, 105,008 were paid authorizations (14.1%). The total expense in the period was BRL 374,089,836. This expenditure is right skewed (R(105,008) = 0.438 p = 0.000 - skewness 41.3) (Figure 1).

The most frequent health care events in the analyzed authorizations were: emergency room consultation (6.1% of the analyzed authorizations contain this event), individual psychotherapy session (5.7%) and RT-PCR screening for COVID-19 (5%). A total of 96.2% of the prior authorizations have no description of ICD-10 and only 587 (0.3%) have ICD-10 B34.2 - “Coronavirus infection, unspecified”.

The clinical indication variable had 64,917 (35.2%) authorizations with only one word or number and 77.6% of authorizations had up to 5 words. After treating the variable, the most common words were “covid” appearing 6,561 times, “pronto” (3,821) and “socorro” (3,692) [emergency room]. The longest sentence was 104 words.

As for treatment type, 90.7% were clinical treatments, 7.8% surgical and 0.3% obstetric. Regarding the health care regime, 81% were outpatient care, 16.9% hospital care, and 1% home care. Inpatient clinical care corresponded to 15,741 authorizations - 8.5% of the total (Table 1).

**Table 1:** Number of prior authorizations analyzed by type of treatment according to supplementary health care provider authorization care regimen. São Paulo, Brazil, September/2019 to June/2022.

Care Regimen	Treatment type									
	Clinical		Surgical		Obstetric		Others		Total	%
	n	%	n	%	n	%	n	%		
Hospitalization	15,741	50.4	13,085	41.9	571	1.8	1,830	5.9	31,227	16.9
Day hospital *	310	16.1	1,208	62.8	5	0.3	400	20.8	1,923	1.0
Home care	1,789	96.0	47	2.5	0	0.0	28	1.5	1,864	1.0
Outpatient clinic	149,351	100.0	1	0.0	0	0.0	4	0.0	149,356	81.0
Emergency room	1	100.0	0	0.0	0	0.0	0	0.0	1	0.0
Total	167,192	90.7	14,341	7.8	576	0.3	2,262	1.2	184,371	100.0

* The day hospital care regimen corresponds to an intermediate care regimen between outpatient clinic care and hospitalization regimen. This service is considered to last up to a maximum of 12 hours.

Regarding the objective of care, 75.1% were for diagnosis and 6.5% reparative treatment - 18.3% of the prior authorizations had no objective of care filled in. In the outpatient regimen, the diagnostic objective was more frequent (80.6%). In the hospitalization regimen, there is an important group of reparative care (34.5%) (Table 2).

**Table 2:** Number of prior authorizations analyzed by treatment objective according to care regimen of the supplementary health care provider authorizations. São Paulo, Brazil, September/2019 to June/2022.

Regimen care	Care objective													
	No information		Diagnostic		Repairing		Palliative		Preventive		Therapeutic		Total	%
	n	%	n	%	n	%	n	%	n	%	n	%		
Outpatient clinic	28,687	19.2	120,384	80.6	43	0.0	22	0.0	3	0.0	217	0.1	149,356	81.0
Home care	93	5.0	1,626	87.2	59	3.2	-	-	-	-	86	4.6	1,864	1.0
Day hospital	463	24.1	393	20.4	1,066	55.4	-	-	-	-	1	0.1	1,923	1.0
Hospitalization	4,435	14.2	15,996	51.2	10,781	34.5	1	0.0	-	-	14	0.0	31,227	16.9
Emergency room	-	-	-	0.0	1	0.0	-	-	-	-	-	0.0	1	0.0
Total	33,678	18.3	138,399	75.1	11,950	6.5	23	0.0	3	0.0	318	0.2	184,371	100.0

In the topics classified as COVID-19, the untreated BERTopic models presented higher average costs per paid authorization - BRL 10,205 in the one with more than 1,000 authorizations and BRL 10,138 in the one with more than 500 authorizations per topic. They correspond respectively to 1.9% (1,987) and 2.3% (2,443) of the authorizations paid and expenses of BRL 20.3 million (5.4% of total expenditure) and BRL 24.8 million (6.6%) respectively. The two models showed a significant number of paid authorizations considered discrepant - 58.8% (61,723) in the BERTopic +1,000 model and 48.3% (50,716) in the BERTopic +500 model (Table 3).

**Table 3:** Models and characteristics of prior authorizations paid according to suspected COVID-19 infection and outliers of authorizations issued by a supplementary health care provider. São Paulo, Brazil, September/2019 to June/2020.

Models	Topics (n)	Outliers *		Suspected COVID-19 infection authorization topics		
		Paid prior authorization (n)	Expenditure (BRL)	n	Paid prior authorization (n)	%	Prior authorizations expenditure (BRL)	%	Average cost per paid prior authorizations (BRL)
Without word treatment									
BERTopic +500	55	50,716	155,110,004	3	2,443	2.3	24,768,350	6.6	10,138.50
BERTopic +1,000	23	61,723	166,228,195	2	1,987	1.9	20,277,859	5.4	10,205.26
Word2Vec	20	0	0	1	1,005	0,5	4,909,189	1.3	4,884.77
With word treatment									
BERTopic +500	51	47,470	189,461,609	4	3,425	3.3	14,019,644	3.7	4,093.33
BERTopic +1,000	13	38,066	82,426,853	2	1,734	1.7	5,241,321	1.4	3,022.68
Word2Vec	20	0	0	3	5,989	5.7	30,072,836	8.0	5,021.35

BERTopic +500 = minimum 500 authorizations per topic; BERTopic +1,000 = minimum of 1,000 authorizations per topic.

Note: the Word2Vec model classifies all authorizations and therefore.

* Outliers correspond to authorizations not classified in topics by the model.

With the treatment of the “clinicalindication” variable, there was an increase in the number of authorizations of suspected cases of COVID infection in the BERTopic model with more than 500 authorizations (to 3.3% of the total authorizations paid) and a decrease in the model with more than 1,000 authorizations (1.7%) followed by a significant reduction in the total expenditure - BRL 5.2 million and BRL 14 million, respectively, when compared to the same models without word treatment, resulting in a decrease in the average costs per authorization in the two models. There was a decrease in the number of prior authorizations considered discrepant - although still high (36.3% in the BERTopic +1,000 model and 45.2% in the BERTopic +500 model) (Table 3).

The treatment of the “clinicalindication” variable substantially modified the indicators of the Word2Vec model. For cases classified as COVID-19, without treatment, this model presented lower numbers for paid authorizations (n = 1,005, 0.5%), total expenditure (BRL 4,909,189, 1.3%) and average cost per authorization (BRL 4,885) than those for the model with word treatment: 5,989 - 5.7%, BRL 30.1 million - 8%, and average cost of BRL 5,021, respectively (Table 3).

The comparison between the 06 models showed that the BERTopic +1,000 model without treatment has a lower number of authorizations classified as suspected covid with high total expenditure and the Word2Vec model with treatment has a higher number of authorizations classified as suspected covid with higher total expenditure (BRL 30 million), but resulting in a lower average cost (Table 3).

The evaluation of the classification quality of the BERTopic +1,000 model shows that, of the first 100 cases analyzed manually, 70 are related to suspicion of or infection by COVID clearly indicated in the text of the clinical indication variable. These patients had expenditure of BRL 11.5 million - 56.5% of the total expenditure identified in this model (Box 2).

**Box 2:** Evaluation of the BERTopic +1,000 model without treatment by manual classification of the 15 authorizations ordered by cost of suspected cases of COVID-19 infection in a supplementary health care provider. São Paulo, Brazil, September/2019 to June/2022.

PRIOR AUTHORIZATIONS	DESCRIPTION OF THE REQUEST FOR PRIOR AUTHORIZATION *	EXPENDITURE PER AUTHORIZATION (BRL)	CLASS **
1	Respiratory distress. I REQUEST THE HOSPITALIZATION OF THE NB IN THEIR OWN CARD BECAUSE THE HOSPITALIZATION PERIOD EXCEEDED THE 30 DAYS IN THE MOTHER’S CARD FROM DEC 19, 2021	709,892	Respiratory disease in newborn/Symptoms
2	severe acute respiratory sd, covid pcr 07 days, diabetes	676,338	COVID-19
3	covid confirmed evolving with hyperemia	650,212	COVID-19
4	COVID INFECTION	515,771	COVID-19
5	flu-like symptoms for 10 days. respiratory distress. With Tachydyspnea	428,903	Respiratory disease/Symptoms
6	reports covid+ comes for evaluation. reports worsening of dyspnea and s02 87 at home	423,112	COVID-19
7	presenting dyspnea respiratory distress 38 fever and drop in saturation	415,292	Respiratory disease/Symptoms
8	COVID FOR 7+ DAYS, CT SHOWS BETWEEN 15 AND 50% OF THE LUNG AREA AFFECTED.	402,072	COVID-19
9	COVID-positive patient presenting dyspnea at medium exertion and 88% oxygen saturation on room air	390,321	COVID-19
10	Microorganism pneumonia	387,281	Respiratory disease/Symptoms
11	COVID+ patient with worsening respiratory symptoms in the last 24 hours	382,854	COVID-19
12	COVID-19 VIRAL PNM?	378,524	COVID-19
13	BCP, COVID	352,845	COVID-19
14	EXTREME PRETERM NEWBORN, CHILD OF COVID POSITIVE MOTHER, HOSPITALIZED IN NEONATAL ICU REQUIRING VENTILATORY, CLINICAL AND HEMODYNAMIC SUPPORT. I REQUEST HOSPITALIZATION OF NB BECAUSE MOTHER WAS DISCHARGED FROM HOSPITAL AND NB NEEDS TO REMAIN HOSPITALIZED FOR SUPPORT AND TREATMENT.	337,206	Respiratory disease in newborn/Symptoms
15	COVID-19 PATIENT EVOLVING WITH DECREASED SATURATION AND DYSPNEA REQUIRING O2	331,786	COVID-19
16	COVID ? Shortness of breath, dry cough for ten days with worsening for two days CT with 25-50 affected	309,762	COVID-19
17	Patient with myalgia, dry cough and fever for 7 days. 05 days ago was tested for COVID-19 with a positive result. Fever since then. Already using azithromycin	302,743	COVID-19
18	Patient on D14 of COVID-19-positive symptoms, admitted to ER desaturating. Was admitted to emergency room due to clinical condition, with complete monitoring and 5l/min O2 catheter. Evolving with improving saturation. Chest CT: areas of ground-glass opacities and consolidation dispersed in both lungs, of peripheral distribution.	281,346	COVID-19
19	Hypertensive and hepatopathic patient with COVID-19 infection, progresses with clinical worsening of cough, dyspnea and desaturation.	276,431	COVID-19
20	COVID+ FOR 07 DAYS, DYSPNEA AND WORSENING SATURATION	270,206	COVID-19
21	B972-CORONAVIRUS, AS CAUSE OF DISEASES CLASSIFIED IN OTHER CHAPTERS J180-BRONCOPNEUMONIA NOT SPECIFIED	242,635	COVID-19
22	COVID-POSITIVE	229,489	COVID-19
23	COVID-positive patient, with onset of symptoms 10 days prior, reports progressive dyspnea and cough for 5 days	226,263	COVID-19
24	Bronchodysplastic patient + with SMA 1, with tracheostomy and gastrostomy, in bronchospamum associated with hypoxemia. ICU admission due to the need for mechanical ventilation.	224,587	Respiratory disease/Symptoms
25	COVID-19 INFECTION	215,832	COVID-19
26	COVID-19 POSITIVE PATIENT EVOLVES WITH WORSENING TRANSFERRED TO ICU	203,934	COVID-19
27	COVID-POSITIVE	198,838	COVID19
28	Transfer of [redacted] with bed assigned for hospitalization 8th day of symptoms: cough, headache, myalgia, dyspnea.... yesterday reports having had the worst day since the onset of symptoms with more pronounced dyspnea, which is why returned to seek medical care On Tuesday Dec 8 had already attended this UPA and underwent PCR for COVID (still without result) and underwent chest CT (reports that the examination was normal and therefore was released for treatment at home)	193,848	COVID-19
29	nb with respiratory distress	181,064	Respiratory disease in newborn/Symptoms
30	COVID-positive	180,297	COVID-19
31	covid pneumonia with secondary infection, fever and yellowish sputum. desaturation. ct with mild extensive imaging	177,360	COVID-19
32	PNEUMONIA + COVID ?	167,661	COVID-19
33	Suspected COVID-19	162,645	COVID-19
34	DYSPNEA + COUGH + FEVER + LYMPHOPENIA - WITH SUSPECTED COVID-19	156,417	COVID-19
35	BCP, COVID-19 DYSPNEA, DECREASING O2 SAT, ROOM AIR - 91-92% ROOM AIR. CT SCAN SHOWS SIGNIFICANT EFFUSION MORE ON THE RIGHT SIDE, WITH DIFFUSE INFLT CARD SINUS, PERIPH	143,477	COVID-9
36	Confirmed contact with COVID-19 Progresses with cough, dyspnea and fever	142,496	COVID-19
37	COVID + 09/03 present fever, dyspnea, headache	141,157	COVID-19
38	COVID-19-positive, patient with worsened general condition	139,158	COVID-19
39	Patient with confirmed Covid-19 in D7 of symptoms evolves with clinical worsening, dyspnea, drop in saturation and persistent fever	134,264	COVID-19
40	PATIENT REPORTS SHORTNESS OF BREATH AND FEVER FOR 11 DAYS (APR 20 HAD A POSITIVE DIAGNOSIS FOR COVID) HEMOPTYSIS FOR 5 DAYS, WORSENING OF DYSPNEA TODAY. SAT 88%. USE OF AZITHROMYCIN WITH NO IMPROVEMENT. DENIES ALLERGIES.	128,084	COVID-19
41	COVID-19 + with 92% oxygen saturation on RA	122,251	COVID-19
42	COVID+ PCR on Sep 27 onset of symptoms on Oct 25 - D6 of symptoms returns due to cough, weakness, inappetence, diarrhea and vomiting hypertensive, diabetic, overweight denies coronary artery disease	107,067	COVID-19
43	PATIENT COMES TO THE CONSULTATION WITH DYSPNEA AND DESATURATION. NOT VACCINATED FOR COVID (DID NOT WANT TO TAKE THE VACCINE BECAUSE OF BEING AFRAID). HAD PCR 4 DAYS AGO FOR COVID (POSITIVE). TODAY BEGAN INTENSE DYSPNEA AND DESATURATION. PHYSICAL EXAMINATION SHOWS GROSS CREPITUS IN RHT AND DIFFUSE WHEEZING. PRESENTS WITH MOTOR DEFICIT SECONDARY TO PREVIOUS ISCHEMIC STROKE.	105,904	COVID-19
44	PATIENT WITH DRY COUGH AND MALAISE FOR 1 WEEK, DIAGNOSED WITH COVID 4 DAYS AGO VIA PCR. TODAY, COMPLAINING OF DYSPNEA, VENTILATORY DEPENDENT THORACIC PAIN WITH WORSENING COUGH, IN ADDITION TO DIARRHEA.	102,470	COVID-19
45	Patient with dyspnea, O2 saturation drop to 92-94%, wife diagnosed with COVID-19 and hospitalized. Chest CT: 10% of lung affected with ground-glass opacity	99,690	COVID-19
46	DESATURATION + DYSPNEA + COVID-POSITIVE	95,268	COVID-19
47	Bacterial pneumonia	95,260	Respiratory disease/Symptoms
48	PATIENT WITH DRY COUGH, DYSPNEA AND DIFFUSE MYALGIA FOR 5 DAYS, WITH WORSENING IN THE LAST 24 HOURS. CHEST CT WITH GROUND GLASS INVOLVEMENT OF 25-50%, BILATERAL, ASSOCIATED WITH INCREASED CRP (16.9).	88,353	Respiratory disease/Symptoms
49	respiratory failure	85,981	Respiratory disease/Symptoms
50	PATIENT DIAGNOSED WITH COVID-19 IN D5 OF SYMPTOMS, RETURNS WITH WORSENING DYSPNEA, SUB-FEVER STATE AND DESATURATION	82,720	COVID-19
51	Patient with respiratory distress, fatigue on minimal exertion, edema for a day and progressive worsening	82,356	Respiratory disease/Symptoms
52	RESPIRATORY SD TBC COVID?	82,310	COVID-19
53	Bedridden patient with stroke sequelae and dementia undergoing treatment at home for bronchopneumonia presented with worsening clinical desaturation requiring a non-rebreathing mask. ICU hospitalization.	80,711	Respiratory disease/Symptoms
54	low-weight nb with respiratory distress	80,034	Respiratory disease in newborn/Symptoms
55	PATIENT COMPLAINING OF INAPPETENCE FATIGUE , FEVER, TESTS SUGGEST COVID-19	78,061	COVID-19
56	Catheter Inf?COVID?	76,414	COVID-19
57	PNEUMONIA WITH SUSPECTED COVID-19	74,352	COVID-19
58	Respiratory failure. COVID	73,550	COVID-19
59	Patient with dyspnea, drop in o2 saturation to 92-94% with wife diagnosed with covid 19 and hospitalized. Chest CT: 10% of lung affected with ground-glass opacity	72,050	COVID-19
60	Bedridden patient, totally dependent for ABVD (home care), HVA for 3 years, DM, prostate CA for 8 years. Brought by removal from home with a history of drop in arterial BP, fever starting today, tremors, oliguria and coluria. Shortly after presenting respiratory distress with drop in saturation. BP 70/40.	71,589	Respiratory disease/Symptoms
61	PATIENT HOSPITALIZED WITH COVID 19 POSITIVE + DYSPNEA + O2 DEPENDENT + RESPIRATORY DISTRESS. ATTACHED DOCUMENTATION.	70,420	COVID-19
62	bacterial pneumonia in elderly patient	70,373	Respiratory disease/Symptoms
63	52yrs, reports diagnosis of COVID+ 11 days ago (pharmacy test sic) does not bring the result. today, reports frequent dry cough from an early time. Denies fever, diarrhea and shortness of breath. At entry, sat 95% RA with RR 30 bpm and HR 104 bpm BP 160/100 PA HAS denies DM asthma SMK (quit SMK 35yrs ago)	69,982	COVID-19
64	PREMATURE NEWBORN, FIRST TWIN, PRESENTED WITH RESPIRATORY DISTRESS SYNDROME. GASTRIC PERFORATION, SUBMITTED TO EXPLORATORY LAPAROTOMY. ON MECHANICAL VENTILATION, INDICATED TO KEEP HOSPITALIZATION IN A NEONATAL ICU FOR INTENSIVE CARE	69,078	Respiratory disease in newborn/Symptoms
65	COVID19 SAH / DM // COUGH AND LOSS OF APPETITE 4 DAYS AGO , UNMEASURED FEVER// PRESENTED NAUSEA AND HYPOTENSION (100 X 60) WITH SWEATING DURING THE EXAMINATION	68,200	COVID-19
66	patient with laryngotracheomalacia and galenic anuerysm presenting worsening of stridor, and tachydyspnea. On physical examination RR 79, HR 143, lowered liver and bruised skin. Denies fever and other symptoms. Due to possible heart and respiratory failure, I choose to admit to the ICU for better clinical stability.	63,717	Other diseases with respiratory symptoms
67	RESPIRATORY DISTRESS	62,369	Respiratory disease/Symptoms
68	anaphylactic shock (reaction to hydrochlorothiazide administration), desaturation, respiratory failure	61,023	Other diseases with respiratory symptoms
69	PATIENT WITH COVID-19 AND ALTERATIONS IN TOMOGRAPHY, HOSPITALIZED ON AUG 21	60,471	COVID-19
70	COVID-19 FOR 5 DAYS, PROGRESSES WITH PROGRESSIVE DYSPNEA AND INTENSE MYALGIA, HISTORY OF LUPUS AND DVT, USES MAREVAN, CT SHOWS 22% INVOLVEMENT OF PULMONARY PARENCHYMA, ARTERIAL BLOOD GAS WITH S O2=77.6, PCO2=47 MMHG, PO2=42 MMHG, I REQUEST ADMISSION TO OUTPATIENT WARD	59,406	COVID-19
71	HOSPITALIZE AFTER 30 DAYS IN THE PRETERM NB CARD VERY LOW WEIGHT RESPIRATORY DISTRESS SYNDROME HOSPITALIZATION REFERRING TO THE DATE SEP 24, 2021	59,192	Respiratory disease in newborn/Symptoms
72	NEWBORN CESAREAN SECTION, IG 39+6, NEGATIVE SEROLOGIES, GBS NEG, BI, APGAR 6/8, WAS BORN WITH WEAK CRY, CYANOTIC, SAT 71-75% IN O2, INHALED O2, EVOLVES WITH RESPIRATORY DISTRESS BSA 5. REQUIRES HOSPITALIZATION IN NEONATAL ICU	59,002	Respiratory disease in newborn/Symptoms
73	COVID-19 INPATIENT	57,447	COVID-19
74	COVID-19+ patient	56,930	COVID-19
75	Bed granted, pneumonia patient came from [redacted] for suspected bacterial pneumonia	55,078	Respiratory disease/Symptoms
76	Patient's husband with COVID since Dec 04 started with shortness of breath, cough with secretion, coryza and myalgia for 3 days, denies fever. Saturation at home between 85-90%.	53,949	COVID-19
77	COVID pneumonia patient with worsened symptoms	52,159	COVID-19
78	COVID+ (d9 symptoms) with worsening of fever and dyspnea for 3 days o2 sat ra 85% rr 22 sat w/ 02 91% mv + ec diffuse. admission to ICU	51,201	COVID-19
79	Patient referred from [redacted] where was in rehabilitation for stroke sequelae, was admitted to [redacted] on Dec 02, complaining of respiratory distress, fever, DLC. After evaluation by the medical team, imaging and laboratory tests were performed, and Pulmonary Focus Sepsis was evidenced, and hospitalization was chosen for treatment with antimicrobials. Application of 4 bottles of 100UI of Botox is foreseen.	48,812	Respiratory disease/Symptoms
80	COVID in patient in risk group	48,721	COVID-19
81	RESPIRATORY FAILURE PCR COVID+ PA: DEPRESSION	48,307	COVID-19
82	PATIENT WITH COUGH, ADYNAMIA, INAPPETENCE AND DYSPNEA FOR 1 WEEK WITH WORSENING SINCE YESTERDAY ON ADMISSION O2 SAT 92%. LABORATORY WITH INCREASED CRP, LEUKOCYTOSIS WITH DEVIATION 9% RODS, CHEST CT PULMONARY CONSOLIDATION OCCUPYING ALMOST THE ENTIRE LEFT UPPER LOBE (LOBAR PNEUMONIA). COVID-19 EXCLUDED. I REQUEST ADMISSION TO MEDICAL CLINIC OUTPATIENT WARD - LOBAR PNM	47,900	COVID-19
83	************************** I REQUEST RETROACTIVE HOSPITALIZATION FROM DEC 31, 2021 ************************ BEDRIDDEN PATIENT, GTT - TQT FOR 1 DAY WITH DETERIORATED GENERAL CONDITION, FEVER, CHANGE IN URINARY ASPECT, DESATURATION, BRADYCARDIA SIC ........... COVID CONTACT	47,479	COVID-19
84	CONFIRMED COVID-19 PATIENT, PRESENTING WITH CHEST CT WITH INVOLVEMENT > 50% 02 SAT 90% RA AND RR 24.	46,194	COVID-19
85	COVID-POSITIVE PATIENT, WITH DYSPNEA WITH PROGRESSIVE WORSENING, WITH LOW O2 SATURATION. WAS ADMITTED WITH HYPOTENSION, WITH IMPROVED BP AFTER VOLEMIC EXPANSION	45,256	COVID-19
86	Patient for 05 days has difficulty feeding cough and diarrhea. Reports respiratory distress associated with the condition.	45,217	Respiratory disease/Symptoms
87	previously healthy patient with no morbid history, evolving with respiratory symptoms for 8 days	45,137	Respiratory disease/Symptoms
88	PATIENT WITH SHORTNESS OF BREATH, TIREDNESS, HYPOREXIA, MYALGIA AND INTENSE PROSTRATION, COVID TEST + CHEST CT PRESENTS PULMONARY INVOLVEMENT 25% SAT 95%	43,496	COVID-19
89	confirmed covid/sepsis protocol	43,196	COVID-19
90	Patient on day 11 of symptoms, with positive PCR for COVID, presenting gradual worsening with severe fatigue, dyspnea, cough and O2 sat: 68% on RA	42,553	COVID-19
91	patient on day 11 of COVID, evolving with malaise, fatigue, dyspnea and desaturation has a chest CT with 50% involvement	42,353	COVID-19
92	A 78-year-old patient comes to the ER with Pneumonia + Delirium, reports that yesterday was fine, however, during the night had a bad cough, did not sleep well, is more confused and apathetic, reports a feeling of heaviness in head.	41,929	Respiratory disease/Symptoms
93	VIRAL PNM/ COVID 19? I REQUEST AUTHORIZATION FOR RETROACTIVE HOSPITALIZATION - FORM: 2022909741 WITH ZERO DAILY RATE (PATIENT HOSPITALIZED ON DEC 24, 2020)	41,357	COVID-19
94	RETROACTIVE HOSPITALIZATION (APR 13) - PATIENT POSITIVE FOR COVID FOR 14 DAYS WITH PROSTRATION. *** REQUESTED AGAIN BECAUSE DAILY RATE RELEASED WE HAVE NO CONTRACT**	41,061	COVID-19
95	Patient referred from [redacted] where was in rehabilitation for stroke sequelae, was admitted to [redacted] on Dec 02, complaining of respiratory distress, fever, DLC. After evaluation by the medical team, imaging and laboratory tests were performed, and Pulmonary Focus Sepsis was evidenced, and hospitalization was chosen for treatment with antimicrobials. Evolution/intercurrences: Clinically and hemodynamically stable, less secretion.	40,420	Respiratory disease/Symptoms
96	RESPIRATORY DISTRESS	40,263	Respiratory disease/Symptoms
97	POST COVID LEFT CALF PAIN AND CRAMPING	39,357	COVID-19
98	PTNB, AGA, RESPIRATORY DISTRESS REQUIRING HOSPITALIZATION FOR VENTILATORY SUPPORT AND TRANSITION TO ENTERAL DIET	38,498	Respiratory disease in newborn/Symptoms
99	PATIENT HOSPITALIZED ON APR 08 DISCHARGED ON APR 19 IN ISOLATION DUE TO COVID-19 - I REQUEST HOSPITALIZATION WITH A RETROACTIVE DATE DUE TO CHANGE IN RELEASED CODE	38,284	COVID-19
100	Patient HAS + DM + DLP + COVID confirmed on Aug 19, 2020, onset of symptoms on Aug 16, 2020. Came to ER with complaint of shortness of breath + diarrhea + myalgia and fever starting today. In the screening SatO²:95%.	38,254	Respiratory disease/Symptoms

* The description of the request for prior authorization has not been changed and is indicated as it is in the request;

** Author classification based on analysis of the “clinicalindication” field. The classification was independent of the classification generated by the model. Cases classified as COVID-19 indicate suspected infection of a patient whose previous authorization was issued under the terms contained in the clinical indication.

Other 20 patients have signs, symptoms or respiratory diseases that may or may not be related to COVID. The expenditure in this group was BRL 2.5 million. Other 8 cases are of newborns with respiratory distress all with no connection to the disease except one extreme newborn born to a mother with COVID. The other 2 cases present respiratory signs and symptoms unrelated to the disease (Box 2). Box 3 shows the first 15 authorizations of this quality assessment with the original description of the prior authorization, the respective manual classification and expenditure per authorization. The analysis of the first 100 cases is shown in the Box 2.

**Box 3:** Evaluation of the BERTopic +1,000 model without treatment by manual classification of the 15 authorizations ordered by cost of suspected cases of COVID-19 infection in a supplementary health care provider. São Paulo, Brazil, September/2019 to June/2022.

PRIOR AUTHORIZATIONS	DESCRIPTION OF THE REQUEST FOR PRIOR AUTHORIZATION *	EXPENDITURE PER AUTHORIZATION (BRL)	CLASS **
1	Respiratory distress. I REQUEST THE HOSPITALIZATION OF THE NB IN HIS OWN CARD BECAUSE THE HOSPITALIZATION PERIOD EXCEEDED THE 30 DAYS IN THE MOTHER 'S CARD FROM DEC 19, 2021	709,892	Respiratory disease in newborn/Symptoms
2	severe acute respiratory sd, covid pcr 07 days, diabetes	676,338	COVID-19
3	covid confirmed evolving with hyperemia	650,212	COVID-19
4	COVID INFECTION	515,771	COVID-19
5	flu-like symptoms for 10 days. respiratory distress. With Tachydyspnea	428,903	Respiratory disease/Symptoms
6	reports covid+ comes for evaluation. reports worsening of dyspnea and s02 87 at home	423,112	COVID-19
7	presenting dyspnea respiratory distress 38 fever and drop in saturation	415,292	Respiratory disease/Symptoms
8	COVID FOR 7+ DAYS, CT SHOWS BETWEEN 15 AND 50% OF THE LUNG AREA AFFECTED.	402,072	COVID-19
9	COVID-positive patient presenting dyspnea at medium exertion and 88% oxygen saturation on room air	390,321	COVID-19
10	Microorganism pneumonia	387,281	Respiratory disease/Symptoms
11	COVID+ patient with worsening respiratory symptoms in the last 24 hours	382,854	COVID-19
12	COVID-19 VIRAL PNM?	378,524	COVID-19
13	BCP, COVID	352,845	COVID-19
14	EXTREME PRETERM NEWBORN, CHILD OF COVID POSITIVE MOTHER, HOSPITALIZED IN NEONATAL ICU REQUIRING VENTILATORY, CLINICAL AND HEMODYNAMIC SUPPORT. I REQUEST HOSPITALIZATION OF NB BECAUSE MOTHER WAS DISCHARGED FROM HOSPITAL AND NB NEEDS TO REMAIN HOSPITALIZED FOR SUPPORT AND TREATMENT.	337,206	Respiratory disease in newborn/Symptoms
15	COVID-19 PATIENT EVOLVING WITH DECREASED SATURATION AND DYSPNEA REQUIRING O2	331,786	COVID-19

Note: the complete table containing the first 100 prior authorizations analyzed is shown in the Box 2.

* The description of the request for prior authorization has not been changed and is indicated as it is in the request;

** Author classification based on analysis of the clinical indication field. The classification was independent of the classification generated by the model. Cases classified as COVID indicate suspected infection of a patient whose previous authorization was issued under the terms contained in the clinical indication.

The traditional method using SQL and selection of prior authorizations containing the words covid, sars, coronavirus and coronavírus resulted in 3,703 authorizations paid with a total expenditure of BRL 23,611,018 - average cost of BRL 6,376.

By comparing the traditional method with the generated NLP models, there are selected prior authorizations not classified by the models, cases of interest that were lost. These authorizations spread across the different topics of the models but concentrated in the topic with outliers, where it is not possible to make the classification.

In the BERTopic models, the greatest loss of cases occurred in the untreated model with more than 1,000 authorizations - 2,377 (64.2%) authorizations were not classified by the model, had a total expenditure of BRL 8.7 million and an average cost of BRL 3,673. The BERTopic model with more than 500 authorizations without treatment was little better - 1,622 (43.8%) unclassified authorizations, expenditure of BRL 5.1 million and average cost per authorization of BRL 3,214. These lost cases have an average cost per authorization almost 3 times lower than those classified by the models. The treatment of the words caused these models to stop classifying the less severe cases, the average costs per authorization of the lost cases were BRL 9,323 and BRL 7,217 in the BERTopic +1,000 and BERTopic +500 models respectively.

On the other hand, the models classified authorizations not selected in the traditional method. The 362 authorizations in excess in the BERTopic +500 untreated model that do not contain the words of the traditional search have an average cost of BRL 17,196 - an expense of BRL 6.2 million. In the BERTopic +1,000 untreated model, prior authorizations with the same characteristic (661 authorizations) have an average cost of BRL 8,165 and a total expense of BRL 5.4 million. The Word2Vec model with the best performance in this regard - 2,703 authorizations with expense of BRL 11,369,283 and average cost per authorization of BRL 4,206 - is the treated model (Table 4).

**Table 4:** Compares models with traditional word selection method in the classification of authorizations issued by a supplementary health care provider. São Paulo, Brazil, September/2019 to June/2020.

Models	Prior authorization classified in the model		Lost when compared to traditional method				Model found but traditional method lost	
	Paid prior autorizations (n)	Expenditure (BRL)	Paid prior authorizations (n)	%	Expenditure (BRL)	%	Paid prior autorizations (n)	Expenditure (BRL)
Without word treatment								
BERTopic +500	2,081	18,543,341	1,622	43.8	5,067,677	21.5	362	6,225,009
BERTopic +1,000	1,326	14,880,970	2,377	64.2	8,730,048	37.0	661	5,396,889
Word2Vec	985	4,842,603	2,718	73.4	18,768,415	79.5	20	66,586
With word treatment								
BERTopic +500	2,249	13,117,398	1,454	39.3	10,493,620	44.4	1,176	902,247
BERTopic +1,000	1,730	5,217,648	1,973	53.3	18,393,370	77.9	4	23,674
Word2Vec	3,286	18,703,552	417	11.3	4,907,466	20.8	2,703	11,369,284

BERTopic + 500 = minimum 500 authorizations per topic; BERTopic + 1,000 = minimum of 1,000 authorizations per topic.

Traditional method: *structured query language* (SQL) considering the presence of uppercase and lowercase words *covid*, *sars*, *coronavirus* and *coronavírus*.

Note: the traditional method found 3,703 authorizations with a total expenditure of BRL 23,611,018.

The BERTopic models generated other topics of interest - related to cancer (1,500 prior authorizations and BRL 6,662,411 spent), orthopedic diseases (4,531 prior authorizations and BRL 13,675,723 spent) and mental illnesses (3,603 prior authorization and BRL 818,893 spent). These topics vary depending on the method employed - the BERTopic +1,000 models, treated or untreated, were worse generating few additional topics. The topics formed by each model are shown in the Box 4, 5, 6 and 7.

**Box 4:** Number of prior authorizations by topics generated by the BERTopic +500 model without word treatment and respective description of the authors.

BERTopic +500 MODEL TOPICS WITHOUT TREATMENT		
TOPIC	NUMBER OF PRIOR AUTORIZATIONS IN THE TOPICS GENERATED BY THE MODEL *	TOPICS GENERATED BY THE MODEL **	DESCRIPTION OF THE RELEVANT TOPICS ***
-1	85,505	-1_icd_of_in_treatment	
0	13,551	0_knee_right_left_injury	Right or left knee injury
1	8,848	1_routine_check_up_heartburn	Routine exams/check-up
2	8,567	2_extension_05_01_03	Administrative routine
3	7,783	3_exam_sroutine_serological_eroin	Routine exams/check-up
4	4,663	4_exam_oct_xray_bhcg	
5	4,032	5_patient_with_cough_condition	Patient with cough
6	3,668	6_covid_19_with_symptoms	With symptoms of COVID-19
7	2,883	7_mg_ev_continuity_cycle	
8	2,713	8_icd_10_e66_m54	
9	2,324	9_f41_f410_f415_	ICD-10 (group of mental and behavioral disorders)
10	2,213	10_clarify_esophagitis_mixed_clarify	
11	1,779	11_attachment_in_ema_pm	
12	1,479	12_f33_f34_fr33_depression	ICD-10 (group of mental and behavioral disorders)
13	1,290	13_abdominal_vomiting_pain_nausea	Abdominal pain with nausea and vomiting
14	1,265	14_emergency_room_adult_emergency	
15	1,253	15_arterial_vascular_cerebral_tachycardia	
16	1,240	16_m54_m51_m65_m75	ICD-10 (group of musculoskeletal diseases)
17	1,221	17_therapy_terpia_teraoia_teratoma	
18	1,081	18_intratension_routine_exams	
19	1,073	19_f32_f53_f328_f323	ICD-10 (group of mental and behavioral disorders)
20	1,048	20_location_in_patient_md224	
21	1,022	21_cough_fever_myalgia_coryza	Fever with coryza, cough and myalgia
22	1,012	22_endoscopy_colonoscopy_polypectomy_colon	Endoscopy/Colonoscopy with polypectomy
23	1,012	23_pains_pain_arrhythmia_pateolo	
24	997	24_pain_tbc_ver_abdominal	Abdominal pain to be clarified
25	963	25_individual_psychotherapy_week_2x	Individual psychotherapy
26	961	26_malignant_neoplasm_breast_tumor	Malignant neoplasm of breast
27	960	27_attachment_order_as_physician	
28	891	28_ico_atc2014_uniarterial_atc	
29	876	29_routine_screening_exam	Routine exams/check-up
30	871	30_evaluation_hippotherapy_physician_clinical	
31	838	31_i10_oncological_oncological_patient	Cancer patient (ICD-10 hypertension)
32	781	32_cervical_cervicalgia_lumbar_spine	Spine orthopedics
33	750	33_annual_vaccine_quadruple_vaccine	Annual vaccination
34	748	34_attachments_see_metastasis_followup	
35	732	35_hematuria_double_ureterolithiasis_ureter	Hematuria with ureterolithiasis
36	732	36_auditor_validation_as_physician	Administrative routine
37	693	37_flu-like_flu_symptoms_flu_days	Flu symptoms
38	690	38_psychotherapy_psychology_individual_individual	Individual psychotherapy
39	689	39_covid_self_esteem_low	COVID-19
40	668	40_lumbago_lumbago_discogenica_lumbasciatalgia	Spine orthopedics
41	662	41_consultation_emergency_room_in	Emergency room consultation
42	661	42_has_h40_cataract_psychotherapy	
43	659	43_ps_patient_in_psa	
44	656	44_attachment_f10_f19_f103	
45	635	45_hernia_disc_hernia_disc	Spine orthopedics
46	567	46_there_is_no_not	
47	560	47_individual_psychotherapy_session_session	Individual psychotherapy
48	544	48_malignant_neoplasia_screening_for	Screening for malignant neoplasm
49	518	49_in_attachment_attachment_40313498	
50	517	50_suspected_h1n1-covid	Suspected COVID-19 or H1N1
51	512	51_trauma_trauma_fall_face	Fall-related face trauma
52	509	52_icd10_f41_anxious_others	
53	506	53_rotator_cuff_syndrome_impact	Rotator cuff syndrome
54	500	54_technical_pertinence_monitor_d22	Administrative routine

ICD-10: International Classification of Diseases, 10th revision.

Note: topic -1 is considered “outlier” according to the model.

* Includes all authorizations including zeroed values;

** Topics automatically generated by the model;

*** Qualitative analysis of the name generated by the topic by the authors.

**Box 5:** Number of prior authorizations by topics generated by the BERTopic +500 model with word treatment and respective description of the authors.

BERTOPIC + 500 MODEL TOPICS WITH TREATMENT		
TOPIC	NUMBER OF PRIOR AUTORIZATIONS IN THE TOPICS GENERATED BY THE MODEL *	TOPICS GENERATED BY THE MODEL **	DESCRIPTION OF THE RELEVANT TOPICS ***
-1	84,133	-1_flu_disorder_disorders_right	
0	38,255	0_nan_dd_pd_snc	
1	5,705	1_day_authorization_day_icu	Administrative routine
2	4,957	2_knee_right_left_fracture	Left or right knee fracture
3	3,208	3_f41_f52_f42_f10	ICD-10 (group of mental and behavioral disorders)
4	2,692	4_just_vie_ee_ah	
5	2,323	5_icde039_icdm224_icdi25_icdi839	
6	1,990	6_covid_test_covide_sepsis	Testing for COVID-19 and sepsis
7	1,971	7_emergency_room_adult_emergency	Emergency room consultation
8	1,746	8_f33_f34_fr33_ffffff33	
9	1,738	9_arterial_angina_vascular_syncope	
10	1,585	10_cervical_spine_lumbar_cervicalgia	Spine orthopedics
11	1,438	11_abdominal_nausea_vomiting_nega	
12	1,409	12_neoplasia_malignant_tumor_breast	Malignant neoplasm of breast
13	1,399	13_f32_corona_vírus_icdb342	COVID-19
14	1,294	14_pains_intense_eyes_pain	Eye pain
15	1,288	15_renal_hematuria_calculus_double	Kidney stone hematuria
16	1,286	16_suspected_covid_suspected_susp	Suspected COVID-19
17	1,258	17_therapy_rehabilitation_members_sup	
18	1,163	18_endoscopy_colonoscopy_polypectomy_colon	Endoscopy/Colonoscopy with polypectomy
19	1,160	19_covid_positive_contact_cough	Positive test for COVID-19, patient with cough
20	1,149	20_mg_cyclo_ansentron_sc	
21	1,082	21_er_routine_exams_send	
22	1,051	22_icdf41_icdc41_tcg_icdf41p	
23	1,040	23_icdb07_icdm545_icd_injuries	
24	989	24_individual_psychotherapy_medical_clinic	
25	951	25_cough_coryza_dry_throat	Cough, coryza and dry throat
26	910	26_vaccine_h1n1_will_vaccine	
27	889	27_ico_aticdc2014_ic_has	
28	833	28_consultation_emergency_room_orthopedics	Emergency room consultation
29	802	29_evaluation_icdj111_evaluation_phono	
30	795	30_rotator_cuff_disorder_syndrome	Rotator cuff syndrome
31	773	31_psychotherapy_f81_f80_individudal	Individual psychotherapy
32	698	32_i10_so10_ms10_	
33	685	33_icd_icdzoo_icddizziness_icdprobable	
34	684	34_lowbackpain_canelite_oa_acute	
35	659	35_macular_visual_retina_acuity	Visual acuity, macular or retinal disease
36	652	36_individual_session_psychotherapy_session	Individual psychotherapy
37	606	37_hernia_disc_discal_umbilical	
38	593	38_icdf84_icdf4¹__	
39	592	39_icdf33_g12_day_	
40	583	40_urgency_psychotherapeutic_urgency_pediatrics	
41	582	41_trauma_head_trauma_fall	Fall-related head trauma
42	563	42_allergologist_icdz10_allergologist_allergolosite	Allergist consultation
43	556	43_malignant_neoplasm_screening_screening	Screening for malignant neoplasm
44	548	44_oncological_oncologic_metastasis_followup	Oncology follow-up and metastasis
45	533	45_monitor___	
46	524	46_consultation_office_hm_office	Outpatient consultation
47	522	47_f84_cardiologic_cardiological_cardiology	Consultation with cardiologist
48	514	48_abdominal_pilates_abdomen_paracentesis	
49	510	49_has_development_global_disorder	
50	505	50_z00_z50_z0_zo	

ICD-10: International Classification of Diseases, 10th revision.

Note: topic -1 is considered “outlier” according to the model.

* Includes all authorizations including zeroed values;

** Topics automatically generated by the model;

*** Qualitative analysis of the name generated by the topic by the authors.

**Box 6:** Number of prior authorizations by topics generated by the BERTopic +1,000 model without word treatment and respective description of the authors.

BERTOPIC +1,000 MODEL TOPICS WITHOUT TREATMENT		
TOPIC	NUMBER OF PRIOR AUTORIZATIONS IN THE TOPICS GENERATED BY THE MODEL *	TOPICS GENERATED BY THE MODEL **	DESCRIPTION OF THE RELEVANT TOPICS ***
-1	104,536	-1_of_icd_in_attachment	
0	11,726	0_with_patient_pain_of	
1	9,206	1_knee_right_left_shoulder	Right or left knee or shoulder injury
2	8,858	2_routine_break_check_up	Routine exams/check-up
3	7,794	3_05_extension_01_03	Administrative routine
4	7,781	4_exam_sroutine_eroina_larynx	
5	4,669	5_exam_lab_exam_oct	
6	3,287	6_covid_19_with_patient	With symptoms of COVID-19
7	2,952	7_mg_ev_continuity_for	
8	2,846	8_icd_10_i10_hd	
9	2,496	9_neoplasia_endoscopy_colonoscopy_colon	Endoscopy/Colonoscopy and colon neoplasia
10	2,326	10_f41_f410_f415_cis10	ICD-10 (group of mental and behavioral disorders)
11	2,196	11_clarify_esophagitis_clarify_elucidate	Esophagitis to be clarified
12	1,802	12_attachment_in_somatization_45	
13	1,479	13_f33_f34_fr33_depression	ICD-10 (group of mental and behavioral disorders)
14	1,330	14_see_routine_exam_attachment	
15	1,303	15_emergency_room_h360_h353	
16	1,224	16_therapy_terpia_teraoia_teratoma	
17	1,197	17_f32_f328_f323_ee	ICD-10 (group of mental and behavioral disorders)
18	1,187	18_m54_m51_m75_m65	ICD-10 (group of musculoskeletal diseases)
19	1,066	19_woman_routine_exams	Routine exams/check-up
20	1,054	20_pain_f43_f51_tbc	
21	1,046	21_location_in_patient_md224	
22	1,010	22_pains_pain_arrhythmia_painscovidd	

ICD-10: International Classification of Diseases, 10th revision.

Note: topic -1 is considered “outlier” according to the model.

* Includes all authorizations including zeroed values;

** Topics automatically generated by the model;

*** Qualitative analysis of the name generated by the topic by the authors.

**Box 7:** Number of prior authorizations by topics generated by the BERTopic +1,000 model with word treatment and respective description of the authors.

BERTOPIC + 1000 MODEL TOPICS TREATED		
TOPIC	NUMBER OF PAS IN THE TOPICS GENERATED BY THE MODEL *	TOPICS GENERATED BY THE MODEL **	DESCRIPTION OF THE RELEVANT TOPICS ***
-1	61,881	-1_psychotherapy_consultation_individual_vaccine	
0	65,341	0_mg_right_left_fever	
1	38,253	1_nan___	
2	3,222	2_f41_f52_f91_f42	ICD-10 (group of mental and behavioral disorders)
3	2,700	3_ok_just_partir_vie	
4	1,991	4_covid_test_covide_sepsis	Testing for COVID-19 and sepsis
5	1,971	5_emergency_room_orthopedics_adult	Orthopedic emergency room consultation
6	1,745	6_f33_f34_fr33_ffffff33	
7	1,381	7_f32_f33_f38_	ICD-10 (group of mental and behavioral disorders)
8	1,289	8_pains_intense_chest_pain	
9	1,286	9_suspected_covid_suspected_family	Suspected COVID-19
10	1,221	10_therapy_terpia_teraoia_therapeutic	
11	1,049	11_icdf41_icdc41_tcg_icdf41p	
12	1,041	12_er_yag_capsulotomy_laser	

ICD-10: International Classification of Diseases, 10th revision.

Note: topic -1 is considered “outlier” according to the model.

* Includes all authorizations including zeroed values;

** Topics automatically generated by the model;

*** Qualitative analysis of the name generated by the topic by the authors.

## Discussion

The BERTopic model without word treatment selected more severe patients while the Word2Vec model with word treatment selected less severe patients. As early as 1998, Hernández & Stolfo ^34^ discussed the difficulty of working with real-world data. This challenge is greater with the use of unstructured data. The 100 cases manually analyzed show differences in how to name the virus, amplified by the peculiarities of the Portuguese language - accents, for example. Another challenge is the breadth of information - most authorizations were filled out with sentences of up to 5 words. Still, the BERTopic model was able to select cases with the description “flu-like symptoms for 10 days. Respiratory distress. With tachydyspnea” as suspected virus infection. It is observed that there is no explicit mention of COVID-19 and while respiratory has accent, tachydyspnea does not, an example of the problem of unstructured data.

This difficulty should explain why there are few studies using NLP applied to early detection of the disease. In a review of the use of artificial intelligence tools applied in the response to the pandemic, Syrowatka et al. ^35^ indicated only 1 NLP-based study for early diagnosis or patient screening. Most studies (65 of 78) used chest image processing techniques. The authors indicate that most studies analyzed are still in the research phase and few are used for decision-making ^35^. A specific review on the use of NLP in the pandemic showed the use of topic modeling applied in the search for literature related to COVID-19 and non-adherence to social distancing with use ^36^.

In a study comparing different topic modeling methods in social media, Egger et al. ^37^ showed that the BERTopic model better separated the topics and its analysis tools enable a better understanding of the interrelations between the topics. Such tools are visual and the authors state that the topics require human interpretation ^37^.

As for human participation, a holistic and multidisciplinary view is needed, based on the human interpretation of the topics (modeling dimension) and the well-being of the patient (health dimension) considering financial aspects (economic dimension).

As an example of the challenge of this holistic view, it is observed that the models studied have opposite behaviors: one selects severe cases and the other selects less severe cases. The implementation of a health promotion program in the context of post-COVID-19 syndrome is much greater than the simple interpretation of the topics generated by an automatic model. It is a multidisciplinary enterprise also comprising the design of the program, identification and correct allocation of patients, their monitoring, evaluation of outcomes and financial results.

Post-COVID-19 syndrome patients require a wide gamut of special care ranging from reestablishment of previous health conditions to rehabilitation ^38^. In this context, it is important to note that automatically generated models and the interpretation of their topics, although interesting, are part of a process that is highly dependent on people. Although, in the health care field, human resources are specialized and expensive, human participation is essential, not only interpreting the topics generated but also designing the entire program in line with this interpretation. It is worth using an NLP model in the early identification of diseases as long as a multidisciplinary team conducts the task of providing patients with quality, accessible and sustainable health care.

Specifically considering the informational dimension, an unsupervised model, especially when there is no word treatment, has some advantages. It is not influenced by the researcher. Another advantage is serving as support for the supervised models being employed as exploratory techniques ^39^. The necessary human interpretation is perfectly consistent in a flow of patient discovery with the following steps: (1) unsupervised exploratory analysis - object of this study; (2) human interpretation and labeling based on the program design; (3) classification of cases; (4) application of labels in a supervised model with discovery of new patients. A supervised model has better performance and direct measures of quality assessment for classification, but the lack of labels on unstructured information makes its applicability very difficult.

In this study, we used two indirect quality assessment methods. In the first, there is human analysis and classification of authorization requests of the BERTopic +1,000 model, selected because of their possible greater severity and simulating the step of classification of cases by specialist. This practical exercise shows the dependence on human interpretation. While most cases (90%) would be of interest for careful evaluation through contact with patient for example, others were clearly misclassified (e.g., “respiratory distress”). However, they are still interesting - one of the cases is a premature newborn from a mother infected by COVID-19 - whose analysis may lead to a specific program for pregnant women in this pandemic period.

The second indirect quality assessment method used *structured query language* (SQL), indicating that BERTopic models lose a significant group of suspected patients. These cases were less severe. The loss was not resolved with a change in the number of documents per topic - there was an increase in outliers - nor with the treatment of words - the groups became less identifiable. These non-classified cases reinforce the need for a semantic context to apply the method that is associated with the quality of the information in the authorization request. Only 25% of prior authoriztions have some information and of these, most have few words, making contextual analysis by the method difficult. There is an old discussion about data quality and its solution in the process of *knowledge discovery in databases* - KDD ^10^. The use of real databases, such as the one used here, has great potential, and can even be used in evidence based on real data provided that the limitations imposed by quality are corrected ^40^ ^,^ ^41^.

The Word2Vec model performed better with word treatment when compared to traditional methods, in part because the treatment involved standardizing the COVID-19 words written in different ways. Although advantageous, this exposes the difficulty of maintaining such a model and it is necessary to consider whether traditional search using SQL would not be better than this model addressed. However, it should be considered that traditional methods for extracting data from texts are subject to human errors, a priori choice of words present in this text requires specialized knowledge ^42^ and may not fully take advantage of real-world information. Traditional database analysis options for identifying patients with certain diseases in providers are limited - ICD-10 are not informed and paid procedures do not allow the identification of the treated disease (e.g., lung computed tomography is paid in the same way for cancers, infections and checkup). There remains access to a wide range of unstructured information in which new methods, even if they need adjustments, can be more effective.

It is observed that, in this real setting with low quality of information, high volume of prior authorizations with missing values or filled in with only one word, the study demonstrated the viability of an unsupervised model for the analysis of prior authorizations from health care providers without any previous treatment with the use of software that is free, easy to use and easy to implement. This type of model is especially useful in the Portuguese language, in which *coronavirus* and *coronavírus* are different words for the computer but with identical meanings. It also addresses phrases such as - “HR: 65BPM RR: 26BPM BP:100/57MMGH SAT: 95% on RA. maintained respiratory distress” because it “understands” that respiratory distress may be related to COVID-19.

Unexpectedly, the model generated other groups of interest. Notably a group of cancer patients in which the topic formed practically describes the diagnosis attributed to patients - “neoplasm, malignant, breast” and groups of patients with orthopedic problems and mental disorders. These are patients who can certainly benefit from health promotion programs.

On the other hand, an unsupervised model selected prior authorizations belonging to cancer patients. This raises serious concerns about the ethical and responsible handling of information. This work highlights the problems that these models can cause in the ethical field ^43^ especially by focusing on the technical application of NLP disregarding the human dimension. There is a need for broad human participation in different stages of the creation of a health promotion program for patients with post-COVID-19 syndrome. This does not make the method less important; it only reinforces the need for human control.

To the best of our knowledge, this is the first study employing this technique using supplementary health care data in Brazil.

### Study limitations

It is a model that cannot be much generalized due to factors such as: (i) being a proprietary base; (ii) difficulty in accessing information due to ethical and legal secrecy; and (iii) the use of the model trained in non-medical corpus in English. We also observed an important amount of authorizations with semantically poor descriptions, impairing the classification. The quality assessment of the model depended on manual analysis by the main researcher, which may introduce a bias that is mitigated by the exposure of the information and its classification.

### Additional studies

The model should be enhanced by supervised method with the inclusion of labels created by specialists. It can also be enriched with other machine learning methods, such as the analysis of the images attached to the authorizations. It is necessary to discuss the ethical aspects of applying automated models, especially when they classify people into disease groups. It is necessary to assess the impact of treatment regimens and objectives (e.g., outpatient and diagnostic) on the behavior of the models. It is necessary to conduct further studies on the interrelation of different dimensions of knowledge and respective professionals in the provision of integrative, collaborative and sustainable care.

## Conclusion

The BERTopic model without word treatment selected more severe patients with suspected COVID-19 infection than the Word2Vec model with word treatment. On the other hand, with word treatment, the latter model was able to select a larger group of suspected cases. It is observed that the decision on the best model depends on the complementary human analysis and on the health promotion program designed.

Compared to traditional methods, it was observed that the BERTopic models did not classify suspected cases, mostly with lower severity, but which may be relevant in an integrated health care model. Thus, it reinforces the exploratory character, its intermediate use for the application of a supervised model and the need to compare results with traditional research methods.

On the other hand, the model also generated topics of interest for future studies, with special attention to suspected cases of cancer patients.

The findings demonstrate the importance of human participation - analysis of the generated topics for correct classification generating information for a supervised model, choice of the best model according to the perspective of health care management (more severe versus less severe patients), design of a health promotion program aligned with this choice and attention to the ethical aspects of the use of machine learning tools in health care.
